# Soft-tissue modification in Class III non-growing patients treated with clear aligners: a prospective clinical trial

**DOI:** 10.3389/fdmed.2025.1584088

**Published:** 2025-04-29

**Authors:** R. Lione, C. Pavoni, F. Gazzani, F. C. De Razza, L. Lugli, P. Cozza

**Affiliations:** ^1^ Department of Health Science, Saint Camillus International University, Rome, Italy; ^2^ Department of Oral and Maxillofacial Sciences, Sapienza University of Rome, Rome, Italy

**Keywords:** soft tissue, Class III, clear aligners, non-growing patient, cephalometric analyses

## Abstract

**Introduction:**

The aim of the present prospective study was to assess the soft tissue modifications induced by camouflage treatment with clear aligners in adult patients with mild Class III malocclusion.

**Methods:**

Patients were consecutively enrolled in the study sample according to the following inclusion criteria: adult age (≥19 years), permanent dentition including second permanent molars, skeletal Class I or slight Class III (−2° < difference between sella-nasion^ point A and sella-nasion^ point B < +2°), normal skeletal divergence pattern (SN^GoGn, 27°-37°), Class III canine and molar relationship at baseline (T0) with moderate lower arch crowding (≤+4 mm), and good compliance with aligners and elastics (≥20 h/day). All the participants underwent the lower distalization protocol, which included extraction of the lower third molars before starting treatment and a 50% sequential lower distalization, and Class III intermaxillary elastics (1/4 in., 6.5 oz) from buttons on the upper first molars to precision cuts at the level of the lower canines to reinforce the anchorage. Lateral cephalograms were taken at T0 (before treatment) and at the end of the clear aligner treatment (T1) with an average time interval of 24 ± 6 months. A customized digitization regimen and cephalometric analysis were created to assess the esthetic outcomes at T1. The significance level was set at *P* < 0.05.

**Results:**

The lower distalization supported by Class III elastics resulted in a slight improvement of the facial esthetic profile with no significant changes in the lower third of the face. A mildly better projection of the upper lip was detected at the end of treatment, mainly due to the extensive use of Class III elastics.

**Discussion:**

The lower distalization supported by Class III elastics determined slight improvement of the facial aesthetic profile with no significant changes in the lower third of the face. A mild better projection of the upper lip was detected at the end of treatment, mainly due to the extensive use of Class III elastics.

## Introduction

The incidence of Class III malocclusions ranges from 0.8% to 12% in the general population with an etiology that can be either genetic or environmental in origin (1). Positional and dimensional disharmony of numerous components of the craniofacial skeleton are involved in Class III malocclusion and for this reason, treating skeletal Class III malocclusions is still one of the biggest challenges in orthodontics (2, 3). Treatment of Class III malocclusion is age- and severity-dependent.

When orthopedic interventions are no longer possible in the case of non-growing patients, orthognathic surgery is often the best treatment option; however, many patients refuse this treatment because of the risks, morbidity, and costs involved (4–6). Class III skeletal malocclusion can be treated with non-surgical orthodontic therapy according to the patient’s requirements when most of the dental and skeletal criteria are favorable for non-surgical treatment. Camouflage treatment can be planned for some Class III malocclusions at the end of growth. The compensatory orthodontic treatment involves displacing teeth relative to their supporting bone to mask an underlying jaw discrepancy with the aim of attaining acceptable occlusion, esthetics, and function (7). In the decision process, soft tissue is one of the most crucial factors and must be analyzed carefully by the orthodontist (8–12). The soft tissues, which include the lips, cheeks, and facial muscles, play a significant role in facial appearance and smile harmony, and are closely related to the relationship between the maxilla and mandible and the consequent dentoalveolar adaptation (13, 14). In the literature, three primary camouflage strategies are described, namely maxillary dentition mesialization, mandibular dentition distalization, and vertical dimension increment. In particular, mandibular distalization increases the lower arch length, recovering the necessary space to correct a Class III relationship (7, 15). Currently, the development of clear aligner technology provides new opportunities, even in the management of complex malocclusion cases, such as Class III discrepancies (13, 14). Several studies (15–19) reported the efficacy of sequential lower molar distalization and Class III elastics in adult patients by means of clear aligners (16–20). However, to our best knowledge, no articles have analyzed the effects of this treatment strategy on the soft tissue. Therefore, the purpose of the present prospective study was to assess the soft tissue modifications induced by camouflage treatment with clear aligners in adult patients with Class III malocclusion.

## Materials and methods

The study was approved by the ethical committee of the Hospital of Rome “Tor Vergata,” (protocol no 75/23) and informed consent was obtained on behalf of all enrolled participants.

### Sample size calculation

A sample size calculation was performed using G*Power software (version 3.1.9.7, Kiel University, Kiel, Germany). With reference to a previous study (8), we determined that a total sample size of 18 participants would be sufficient to detect 2° in the profile facial angle (standard deviation = 2°, alpha = 0.05, power = 0.80).

Patients were consecutively enrolled in the study sample according to the following inclusion criteria: adult age (≥19 years), CS5 vertebral maturation phase [according to the cervical vertebral maturation (CVM) classification], permanent dentition including second permanent molars, skeletal Class I or slight Class III [−2° < difference between sella-nasion^ point A (SNA) and sella-nasion^ point B (SNB) (ANB) < +2°], normal skeletal divergence pattern (SN^GoGn, 27°–37°), Class III canine and molar relationship at baseline (T0) with moderate lower arch crowding (≤+4 mm), and good compliance with aligners and elastics (≥20 h/day). Exclusion criteria included severe skeletal Class III malocclusion (ANB < −2°), transversal maxillary deficiency, extraction treatments other than third molars, and periodontal disease or temporomandibular disorders (TMDs).

All the participants underwent the lower distalization protocol, which included extraction of the lower third molars before starting treatment and a 50% sequential lower distalization with two teeth being distalized at a time. The distalization started with the movement of the lower second molars, followed by the first molars halfway through the process, and so on. Once the canines reached the right position, the “en masse” retraction of the four incisors completed the treatment plan. The protocol comprised the use of Class III intermaxillary elastics (1/4 in., 6.5 oz) from buttons on the upper first molars to precision cuts at the level of the lower canines to reinforce the anchorage during the retraction of the lower premolars, canines, and incisors and to prevent the possible flaring of the incisors during the distalization of the posterior teeth. All patients were asked to wear aligners and Class III elastics for at least 22 h/day with regular clinical checks in the office every 4 weeks. At the end of the distalization, all the patients required a refinement phase, corresponding to the finishing phase, that was performed with a mean number of 19 ± 5 aligners. During the refinement phase, each aligner was worn for 7 days.

Lateral cephalograms were taken at T0 (before treatment) and at the end of the clear aligner treatment (T1) with an average time interval of 24 ± 6 months. All lateral cephalograms at T0 and T1 were standardized to life-size (0% enlargement) (11).

**Figure 1:**
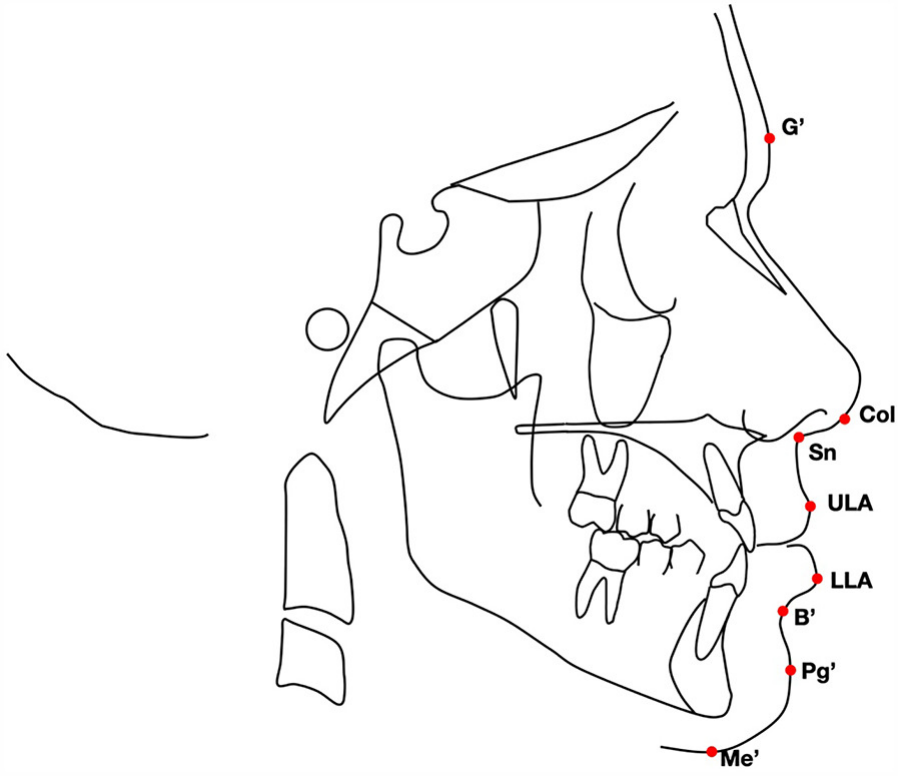
Soft tissue landmarks used in the analysis: G′, soft tissue glabella; Col, columella; Sn, subnasale; ULA, upper-lip anterior point; LLA, lower lip anterior point; B′, soft tissue B point; Pg′, soft tissue pogonion; Me′, soft tissue menton.

**Figure 2:**
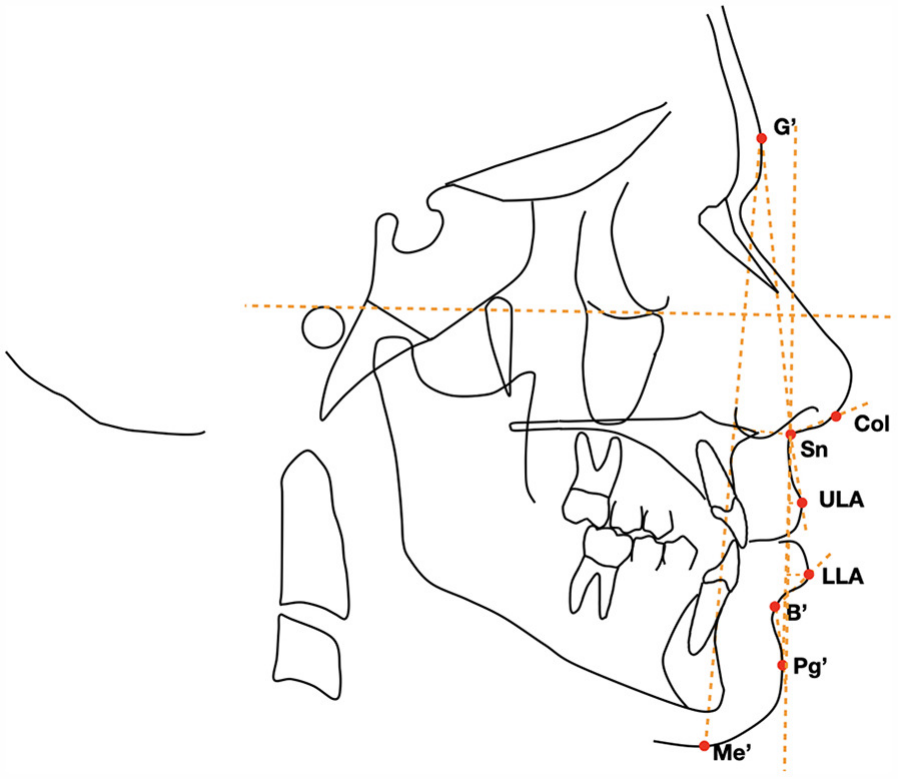
Cephalometric points, lines, and angles used in the analysis: profile facial angle (°), nasolabial (°), lower face (%), upper-lip protrusion (mm), lower-lip protrusion (mm), mandibular sulcus (°), and distance TVL-Pg′ (mm).

A customized digitization regimen and cephalometric analysis provided by Viewbox software (dHAL software, Kifissia, Greece) were performed by the same operator (FG). The customized soft tissue cephalometric analysis (Figure 1), containing measurements using Bergman’s (21), generated six variables: three angular, two linear, and one percentage value. In addition to these soft tissue cephalometric traits, the distance from the soft tissue pogonion to the true vertical line (TVL) was measured using Arnett's analysis (22). All the soft tissue cephalometric measurements are summarized in Table 1 and Figure 2.

**Table 1:** Soft tissue cephalometric variables and their definitions.

Variables	Definition
Profile facial angle (°)	Angle formed by connecting the soft tissue glabella, subnasale, and soft tissue pogonion
Nasolabial angle (°)	Angle formed by the intersection of upper lip anterior and columella at the subnasale
Lower face (%)	Lower third of the face from the subnasale to soft tissue menton, measured vertically and expressed as a percentage of the midface and lower face height, measured from the soft tissue glabella vertically to the soft tissue menton
Lower-lip protrusion (mm)	Perpendicular distance between the lower lip anterior and the subnasale-pogonion line
Upper-lip protrusion (mm)	Perpendicular distance between the upper lip anterior and the subnasale-pogonion line
Mandibular sulcus (°)	Angle formed by the lower lip anterior, soft tissue B point, and soft tissue pogonion when the lips are in repose
Distance TVL-Pg′ (mm)	Distance from the soft tissue pogonion to the true vertical line

**Figure 3:**
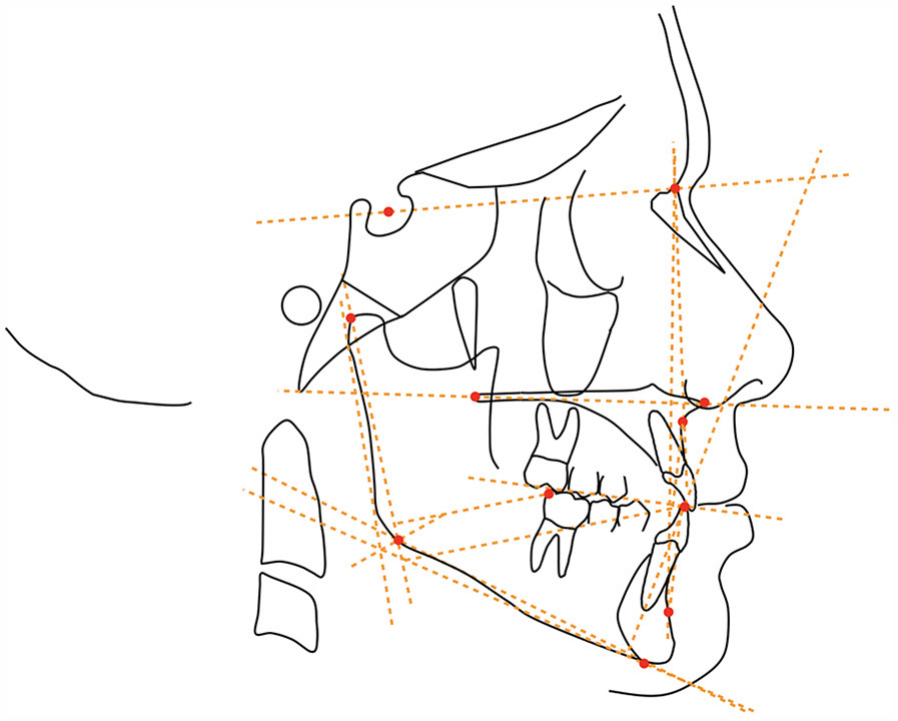
Cephalometric points, lines, and angles used in the analysis: SNA angle (maxillary sagittal position), SNB angle (mandibular sagittal position), ANB angle (maxillomandibular sagittal discrepancy), Wits appraisal (maxillomandibular sagittal discrepancy), SN^MP angle (mandibular plane to the cranial base, upper incisor inclination), IncSup^SN angle (upper incisors to the cranial base), IMPA angle (lower incisors to mandibular plane, lower incisor inclination), overjet (horizontal overlap between the upper and lower incisors), overbite (vertical overlap between the upper and lower incisors), L6-CoGo distance (distance from the lower first molar to the mandibular ramus), and L1-CoGo (distance from the lower first incisor to the mandibular ramus).

Additional cephalometric variables were digitized for each patient at T0 and T1 to provide data on the dento-skeletal correction (Figure 3).

### Statistical analysis

The chi-square test of independence was used to assess differences in gender distribution within the examined group.

T1–T0 differences were assessed by means of the Student's *t*-test for paired data. In the presence of normally distributed data (Kolmogorov–Smirnov test), statistical comparisons were performed with independent sample *t*-tests. The significance level was set at *P* < 0.05. Twelve lateral cephalograms were randomly selected and then traced and measured twice within 2 weeks by the same operator (FG). The measurements from both cephalograms for each patient were analyzed using the intraclass correlation coefficient (ICC). The method of moments was employed to calculate linear measurement errors (23).

## Results

Participant recruitment started in December 2022, while the observation follow-up was conducted until June 2024. Among the 21 patients included in the study group, there were two drop-outs and no deviations from the protocol. The final analyzed sample comprised 19 patients (11 women and 8 men with a mean age of 26 ± .2.6 years). Seven patients presented with a bilateral Class III malocclusion (−2.2 ± 0.5 mm) and 12 patients demonstrated a bilateral end-to-end Class III molar relationship (−1.4 ± 0.5 mm). The duration of active therapy was 24 ± 5.1 months. The patients selected for the study satisfied the compliance criteria of wearing aligners and Class III elastics for at least 20–22 h/day with regular 4-week monitoring. The chi-square test showed no statistically significant difference in gender distribution within the examined group (*P* = 0.49). The intra-observer reproducibility, assessed by calculating ICCs, showed a high level of agreement with variation starting from 0.8° for the facial profile angle and 0.2° for the nasolabial angle. As for the linear measurements, variation starting from 0.3 mm for the upper-lip protrusion and 0.2 mm for the lower-lip protrusion was revealed. The lower distalization protocol and Class III elastics were effective in achieving a Class I molar and canine relationship and the correction of overjet at the end of the active therapy (T1).

The statistical comparisons of the T1–T0 soft tissue changes (Table 2) showed few significant esthetic modifications produced by the distalization protocol.

**Table 2:** Descriptive statistics and statistical comparisons of the T1–T0 soft tissue changes by means of paired samples *t*-tests.

	Treated group (11 women and 8 men—mean age of 26 ± .2.6 years)							
	T0		T1		T1–T0		95% CI of the difference	
Variables	Mean	SD	Mean	SD	Diff.	*P*-value	Lower	Upper
Profile facial angle (°)	144.6	4.8	143.9	3.6	−0.7	NS	−3.435	1.766
Nasolabial (°)	127.1	7.1	125.3	7.5	−1.8	*	−5.970	−3.567
Lower face (%)	51.4	2.8	51.9	3.1	0.5	NS	−0.704	2.369
Upper-lip protrusion (mm)	1.5	1.6	2.6	1.1	1.5	*	0.774	2.337
Lower-lip protrusion (mm)	2.5	0.7	2.2	0.1	−0.3	NS	−0.682	1.553
Mandibular sulcus (°)	141.1	4.7	140.5	4.8	−0.6	NS	−4.459	1.998
Distance TVL-Pg’ (mm)	−2.1	0.4	−2.5	0.5	0.4	NS	−4.202	1.249

SD, standard deviation; Diff., differences; 95% CI, 95% confidence interval; Pg, pogonion; TVL, true vertical line; NS, not significant.

* *P* < 0.05.

Significant but slight differences in the treated group were found for the nasolabial angle (−1.8°), and upper-lip protrusion (+1.5 mm). In contrast, no statistically significant modifications were detected for the profile facial angle and for the soft tissue variables in the lower third of the face (Table 2). Regarding the dento-skeletal effects, no skeletal changes were observed to be significant, while the modifications at the levels of the upper and lower dentition were statistically significant at the end of treatment. The inclination of the upper incisors increased by 2°, while the inclination of the lower incisors was reduced by approximately 3°, thus correcting the initial values of overjet and overbite. The mean distalization of the lower first molar was approximately 1.4 mm (Table 3).

**Table 3:** Descriptive statistics and statistical comparisons of the T1–T0 dento-skeletal changes by means of paired samples *t*-tests.

	Treated group (11 women and 8 men—mean age of 26 ± 2.6 years)							
	T0		T1		T1–T0		95% CI of the difference	
Variables	Mean	SD	Mean	SD	Diff.	*P*-value	Lower	Upper
SNA (°)	80.9	2.3	81.3	1.1	0.4	NS	−1.334	1.188
SNB (°)	80.4	4.2	80.2	1.8	−0.2	NS	−0.415	3.578
ANB (°)	0.5	3.2	1.1	1.6	0.6	NS	−3.932	0.623
Wits (mm)	0.1	2.4	0.5	1.6	0.4	NS	−0.921	1.630
SN^MP (°)	37.5	5.3	36.1	6.9	−1.4	NS	−0.966	3.512
IncSup^SN (°)	106.8	8.3	108.8	4.1	2	*	−8.987	−0.559
IMPA (°)	93.2	4.4	90.3	2.2	−2.9	*	0.704	6.550
Overjet (°)	1	1.5	2.7	0.6	1.6	*	−2.858	−0.888
Overbite (°)	−0.1	1.1	1.4	0.6	1.3	*	−2.289	−0.947
L6-CoGo (mm)	42.9	4.2	41.5	1.7	−1.4	*	1.217	7.607
L1-CoGo (mm)	62.8	3.1	59.9	3	−2.9	*	1.247	6.298

SD, standard deviation; Diff., differences; 95% CI, 95% confidence interval; SNA, sella-nasion^ point A; SNB, sella-nasion^ point; ANB, difference between SNA and SNB; SN^MP, sella-nasion^ mandibular plane; Go, gonion; Co, condylion; L6, lower first molar; L1, lower central incisor; NS, not significant.

* *P* < 0.05.

## Discussion

A balanced soft tissue facial profile has been considered an important outcome to achieve during orthodontic treatment, especially in Class III. However, Yin et al. (24) reported that young adults' subjective perceptions are inaccurate with 78.5% of the participants choosing the straight profile and 17.5% choosing the mild convex profile as the ideal facial profile. Therefore, the principal motive for orthodontic treatment is the improvement of physical attractiveness related to a beautiful smile, rather than deformity of the profile (23). Currently, the analysis of lower molar distalization by means of clear aligners as camouflage treatment in adult patients with Class III malocclusions is still poor in the scientific literature (25).

Rota et al. (19), in a preliminary study, found no changes in the skeletal sagittal and vertical relationship with a mean distalization of the lower first molar of approximately 1.16 mm.

In the present study, all the participants presented with good occlusion at the end of the treatment, with a Class I molar and canine relationship and adequate anterior overjet and overbite, and no signs or symptoms of TMD were revealed during or after the therapy. The lower first molar was distalized with a mean value of 1.4 ± 0.2 mm. The space recovered in the posterior part of the arch allowed for the correction of the occlusal relationship as the patients enrolled in the study were adults and therefore, an increase in mandibular size was not expected during the treatment.

To our best knowledge, no previous studies have analyzed the soft tissue changes at the end of the lower distalization treatment protocol either with clear aligners or by means of conventional fixed appliances (25).

In the present prospective trial, few favorable profile changes were produced at the end of the active phase with clear aligners. The finding of this study showed that the only statistically significant improvements were detected for upper-lip protrusion and nasolabial angle. At the end of treatment, the upper lip became more evident with an increased distance between the upper lip and the subnasal-pogonion line of 1.5 mm. Consequently, the nasolabial angle was statistically significantly decreased by approximately 1.8°. These effects are related to the extensive use of heavy Class III elastics during the treatment. The Class III elastics provided the required anchorage reinforcement for the lower distalization, but at the same time, they resulted in a slight proclination of the upper front teeth, thus producing better support of the upper lip.

No significant differences were found in terms of facial profile angle and in the lower third of the face. In the literature, clear aligners are reported to be effective in restraining the extrusion of posterior teeth during their distalization in the upper and lower arch (16, 26, 27). Furthermore, in this study, there were no significant changes in the vertical dimension at T1. The bite-block effects of the plastic coverage might have accounted for the lack of mandibular clockwise rotation, which is one of the camouflage strategies for a prominent chin in adult patients.

### Limitations

A limitation of this study was the need for patient compliance, which could have resulted in heterogeneity in the results. However, this increased the possibility of extrapolating the results to the ordinary clinical routine. Moreover, the sample size may not be adequate for some variables, as it was calculated considering the ANB angle as the primary variable. In addition, the short-term nature of the study represents a further limitation, as it may not allow for a comprehensive evaluation of the long-term stability and effectiveness of the observed outcomes. Future studies with longer follow-up periods are necessary to validate these results and better assess their persistence over time.

## Conclusions

A lower distalization protocol utilizing clear aligners is a valid therapeutic option for the correction of mild dento-skeletal Class III malocclusions in adult patients. However, the lower distalization protocol supported by Class III elastics resulted in a slight improvement of the facial esthetic profile with no significant changes in the lower third of the face. A mildly better projection of the upper lip was found at the end of the treatment, mainly due to the extensive use of Class III elastics.

## Data Availability

The original contributions presented in the study are included in the article/Supplementary Material, further inquiries can be directed to the corresponding author.
